# The Role of Yeasts and Lactic Acid Bacteria on the Metabolism of Organic Acids during Winemaking

**DOI:** 10.3390/foods9091231

**Published:** 2020-09-03

**Authors:** Ana Mendes Ferreira, Arlete Mendes-Faia

**Affiliations:** 1 University of Trás-os-Montes and Alto Douro, 5001-801 Vila Real, Portugal; afaia@utad.pt; 2 WM&B—Wine Microbiology & Biotechnology Laboratory, Department of Biology and Environment, UTAD, 5001-801 Vila Real, Portugal; 3 BioISI—Biosystems & Integrative Sciences Institute, Faculty of Sciences, University of Lisboa, 1749-016 Lisboa, Portugal

**Keywords:** organic acids, yeasts, fermentation, lactic acid bacteria, wine

## Abstract

The main role of acidity and pH is to confer microbial stability to wines. No less relevant, they also preserve the color and sensory properties of wines. Tartaric and malic acids are generally the most prominent acids in wines, while others such as succinic, citric, lactic, and pyruvic can exist in minor concentrations. Multiple reactions occur during winemaking and processing, resulting in changes in the concentration of these acids in wines. Two major groups of microorganisms are involved in such modifications: the wine yeasts, particularly strains of *Saccharomyces cerevisiae*, which carry out alcoholic fermentation; and lactic acid bacteria, which commonly conduct malolactic fermentation. This review examines various such modifications that occur in the pre-existing acids of grape berries and in others that result from this microbial activity as a means to elucidate the link between microbial diversity and wine composition.

## 1. Introduction

Acidity plays a crucial role in many aspects of the winemaking process, since influences taste and mouthfeel perception, red color intensity, the solubility of tartrate and proteins, and the efficiency of fining [1]. Additionally, the lower the pH is, the lower the susceptibility of wines to microbial spoilage. Acidity is dependent on several factors, such as grape-vine cultivar, climate conditions, and vineyards cultural practices. To assess the best time for harvesting, the amount of acids and sugars in grape berries should be perfectly balanced, always taking into account the type of wine to be produced. In high acidic wines, the major concern is the choice of a deacidification process, and generally, biological deacidification is the preferred approach. In contrast, in low acidic wines, tartaric acid is commonly used for pH adjustment [1,2], mostly because yeast and wine bacteria are unable to metabolize it, while most of the other organic acids can serve as substrates for such microorganisms, contributing themselves, favorably or unfavorably, to the quality of the wine. Thus, a good understanding of acids modification during the winemaking process is essential to make good wines. 

## 2. Organic Acids of Grape Juice

Grape juice largely consists of water, approximately 80%, and many dissolved solids, including organic and inorganic compounds. Next to sugars, the organic acids found in grapes are the second largest group of compounds accounting for nearly 1% of solids present in grape juice [1]. l-tartaric and l-malic acids account for over 90% of the acid content, whereas others, such as citric and ascorbic acid, are present in a lesser extent, representing less than 10% of the total acidity [1]. Grapes are one of the few fruits that contain l-tartaric acid, ranging from 4.5 to 10 g L^−1^, being the predominant acid near grape maturity. It is present as a free acid and as a salt, potassium bitartrate, an important constituent that ensures a suitable pH and plays a crucial role in taste, as well as on the physical, biochemical, and microbial stability of wine [1,3]. On the other hand, l-malic acid is commonly found in many fruits and typical ranges between 2 to 6.5 g L^−1^ in ripen grapes [3]. The major acids are synthetized and degraded by different metabolic pathways: l-malic acid is synthesized in fruit, through the carboxylation of phosphoenolpyruvate in cytosol, that originates oxaloacetate (OAA), which through the cytosolic NAD-dependent malate dehydrogenase (MDH) is reduced to malate [4]. The reversibility of this reaction suggests that the cytosolic MDH and the NADP-malic enzyme (ME) are involved in both malate synthesis and degradation during ripening of several fruit species [4], process apparently boosted by high temperatures. The biosynthesis of l-tartaric acid uses l-ascorbic acid as an intermediate [5,6], and its concentration during ripening remains almost constant, despite of berry enlargement [1,3]. Citric acid contributes to the acidity of grape juice in the range of 0.1 to 0.7 g L^−1^ [3]. Others namely gluconic acid, formed by oxidation of glucose, is present in grapes infected with *Botrytis cinerea,* in concentrations ranging from 1 to 2.5 g L^−1^ [3]. Total acidity tends to decrease as sugar content rises; generally, in ripe grapes, acid levels tend to be lower in warm climate regions than in cooler ones, with tartaric acid being the predominant acid due to its higher stability at higher temperatures. Thus, climate change will impact berry composition, particularly on reduction in acidity, in a magnitude yet not completely known, but certainly with deleterious repercussions to overall sensory balance of wine [7].

## 3. Degradation of Organic Acids by Yeasts

During alcoholic fermentation, acids undergo relevant changes directly or indirectly due to the metabolic activity of yeasts. Tartaric acid concentration slowly declines due to the accumulation of ethanol and to the low temperature of wine storage, which decreases its solubility, leading to its precipitation as potassium bitartrate [1]. l-tartaric acid degradation is associated with species of basidiomycete affinity, and rarely utilized by ascomycete yeasts [8]. Even under high cell density (10^10^ CFUs mL^−1^), weak or no degradation of tartaric acid was found for the wine-yeasts tested [9], suggesting that wine-yeasts lack the biochemical pathway for the degradation of the acid [10]. Gluconic acid is not metabolized under fermentative conditions [10], rather is metabolized by flor-yeasts during biological aging [11]. There is also no evidence that *Saccharomyces* strains can effectively transport or degrade extracellular citrate anaerobic conditions or under a high glucose concentration [3]. Although, a non-*Saccharomyces* yeast, *Pichia fermentans*, in co-culture with *S. cerevisiae*, was able to degrade citric acid in a fruit-wine fermentation [12]. Unlike tartaric acid, malic acid is easily degraded by most wine microorganisms. Based on its ability to utilize l-malic acid and other TCA cycle intermediates, yeasts fall into one of two groups: The Krebs (+) group capable of using one or more of the TCA cycle intermediates with no requirement for assimilable sugars [13], which includes *Candida sphaerica* (an anamorph of *Kluyveromyces marxianus*) [14], *C. utilis* [15], and *Hansenula anomala* IGC 4380 [16]. The transport of malic acid and other dicarboxylic acids across their plasma membrane was found to be substrate inducible and subjected to glucose repression [14,15,16,17]. The Krebs (−) group use one or more of the TCA cycle intermediates only in the presence of glucose or other assimilable carbon sources [14]. This group includes *S. cerevisiae, Schizosaccharomyces pombe*, and *Zygosaccharomyces bailii* [18,19], species with variable aptitude to use malic acid. 

In addition to an efficient transport system, yeasts need an effective intracellular malic enzyme (ME) for efficient utilization of malic acid [20,21]. In *Z. bailii* transport is mediated by a carrier protein specific for the L (−) isomer, which is induced by glucose and repressed by fructose [19]. In *Schiz. pombe*, it is actively transported via a proton/dicarboxylate-symport system [22] mediated by malate permease, encoded by the *Schiz*_*MAE1* gene [23], which is activated by glucose but not induced by the substrate [18]. Fermentable carbon sources are required in *Schiz. pombe* [24] and *Z. bailii* [19] to provide the energy necessary for the active transport of the acid. A mitochondrial carrier has been characterized in *S. cerevisiae* [25], although to date there is no evidence that l-malic acid is transported in any way other than passive diffusion, in a non-dissociated form [26] and regulated by the extracellular pH [17]. 

MEs encompass a ubiquitous family of enzymes with multiple isoforms identified in all living organisms, bacteria, plants, and animals [27], and located in different intracellular compartments. In eukaryotes, ME is found in the cytosol, and/or in the mitochondria, or even in the chloroplasts and cytosol in plants [28,29]. ME catalyzes the oxidative decarboxylation of malate to pyruvate, coupled to the reduction of NAD^+^ or NADP^+^ [29,30,31]. Sequence analysis revealed a high degree of similarity of the amino acids [32], but differed with respect to their intracellular location, substrate affinity and specificity, as well as coenzyme specificity. Based on coenzyme specificity and their ability to decarboxylate OAA, MEs fall into three categories: EC1.1.1.38 (NAD^+^-dependent; also decarboxylates OAA to pyruvate), EC1.1.1.39 (NAD^+^-dependent; does not decarboxylate OAA), and EC 1.1.1.40 (NADP^+^-dependent; also decarboxylates OAA). A differential degree of reversibility of the decarboxylation reaction has also been shown in MEs, ranging from absent to 33% OAA decarboxylation activity in chicken liver, to almost complete in human liver [33], or do not catalyze the reductive pyruvate carboxylation in *Arabidopsis thaliana* NADP^+^-MEs [29,34]. ME have been demonstrated in several yeast species, however Polakis and Bartley [35] failed to find it in *S. cerevisiae* grown in glucose. Later, some mitochondrial ME activity, encoded by *MAE1*, was perceived [31]. Doubts still exist regarding the physiological function of Mae1; the enzyme looks not essential since null mutants still grow anaerobically [31], and it cannot readily act in the anaplerotic role of pyruvate carboxylase, once PYC-negative strains failed to grow on glucose [36]. Although it is responsible for the irreversible decarboxylation of l-malic acid to pyruvate during growth on C-2 compounds, ethanol and acetate [30,31], under aerobic conditions. The location and regulation of the mitochondrial ME (encoded by *MAE1*/YKL029C) limits its action in *S. cerevisiae*, and suggests its role for providing NAD(P)H for the biosynthesis of fatty acids and sterols, as well as the delivery of pyruvate for central metabolism [31]. Under anaerobic conditions or in high glucose-medium, when the TCA cycle is not operating as a cycle in *S. cerevisiae*, some cytosolic enzymes similar to those of the TCA cycle could be used as source of biosynthetic precursors [35,37]. 

Three isozymes of malate dehydrogenase have been reported in *Saccharomyces cerevisiae*: the mitochondrial enzyme Mdh1 that catalyzes the interconversion of malate and OAA, in the TCA cycle [38]. The cytosolic Mdh2 and the peroxisomal Mdh3 act in the interconversion of malate and OAA, both in the glyoxylate cycle, and the former also in gluconeogenesis during growth on two-carbon compounds [39,40]. Mdh3 has lower affinity for OAA and it is involved in the reoxidation of NADH, which is produced from fatty-acid β-oxidation [41]. Mdh1 activity was high in yeast cells grown on non-fermentable carbon sources, and decreased in cells grown on glucose [40], suggesting a diminutive contribution of Mdh1 on malate degradation under wine fermentation conditions. The expression levels of MDH1 were comparable under both aerobic and anaerobic conditions in low-glucose medium [42], indicating high rates of respiration under such conditions. Cytosolic Mdh2 catalyze the reversible reaction of OAA to malate, linked to the maintenance of the equilibrium of both acids and respective NAD/NADH cofactor system. Thus, one can suggest that Mdh2 may be involved in the synthesis or degradation of malate from or to OAA, respectively. 

The first report of the ability of *S. cerevisiae* to metabolize l-malic acid during wine fermentation was published in 1966 [43]. That ability was shown to be strain dependent, which reduce 3–45% of l-malic acid pre-existing in grape juice. Efforts to increase that degradation failed even under high cell density conditions [9]. The weak efficiency of *S. cerevisiae* results from the inefficient uptake transport system for the acid and from the low substrate affinity of its ME. However, there have been reports of a strain of *S. paradoxus* RO88 able to degrade 38% and another strain of *S. cerevisiae* able to degrade 18% of the acid [44]. In non-Saccharomyces yeasts, moderate malic acid consumption has been perceived in sequential fermentations with *T. delbrueckii* (20 to 25%) [45], with *Metschnikowia pulcherrima* [46] or with *Issatchenkia orientalis* which reduced about 30% of the malic acid content in wine [47]. Also *C. zemplinina* and *H. uvarum* strains showed some ability to degrade the acid [48]. Inefficiency to metabolize l-malic acid was unveiled in several laboratory strains [10,16] and in some wine yeast strains [9]. Species of the genera *Pichia* and *Candida* may grow on the surface of wine and under such aerobic conditions can utilize 24–38% of l-malic acid [44]. In this case, malic acid is completely oxidized to CO_2_ through the TCA cycle. The most efficient strains are *Schiz. pombe* [49]. and *Schiz. malidevorans*, which can effectively ferment 95–99% of l-malic acid [9] and strains of *Z. bailii* able to degrade 40–100% of this acid [10,50]. Three genetically different *Schiz. pombe* strains, comparable to *S. cerevisiae*, perform effective malic acid degradation during wine fermentation [47], which can be seen as promising ways to reduce wine acidity. The high efficiency of *Schiz. pombe* to metabolize malic acid is due the three enzymes the malate permease (*mae1*), the cytosolic malic enzyme (EC 1.1.1.38, *mae2*), and a mitochondrial malate dehydrogenase enzyme (EC 1.1.1.37). Under fermentation conditions, the cytosolic ME is involved in the degradation of intracellular l-malic acid, while under aerobic conditions, both enzymes, the ME and MDH (encoded by *Schiz*-*MDH*1), play a role in the metabolism of the acid, albeit the Mdh1 only contributes to approximately 10% of its degradation [28]. l-malic acid is oxidatively decarboxylated to pyruvate and a reduced cofactor through the cytosolic NAD^+^/NADP^+^-dependent ME (*Schiz*-Mae2). Pyruvate can be used for biosynthesis purposes [28] or be converted to ethanol and CO_2_ through pyruvate decarboxylase and alcohol dehydrogenase, with the concomitant reoxidation of NADH [50]. Under aerobic conditions, *Schiz. pombe* still may ferment l-malic to ethanol and, to a much lesser extent, it is oxidized to CO_2_ as a TCA intermediate. In *Z. bailii*, malic acid is oxidatively decarboxylated by ME to pyruvate, which is converted into ethanol and CO_2_, but a small amount escapes from ME and is reduced to succinate by fumarase and fumarate reductase [50]. In *S. cerevisiae* and *Schiz. pombe,* ME can react with both malate and OAA, whereas in *Z. bailii* can only decarboxylate malate [50]. In *Schiz. pombe,* NAD^+^ is the ME cofactor [51], while in *S. cerevisiae*, both NAD^+^ or NADP^+^ are used as electron acceptors, with NAD^+^ being favored [35]. Distinct Km values for the MEs have been determined: 3.2 mM for *Schiz. pombe* [49], 10 mM for *Z. bailii* [50], and 50 mM for *S. cerevisiae* [10,51]. To circumvent the inability of *S. cerevisiae* to efficiently consume extracellular malic acid, due to the absence of a malate permease and to high value of Km for ME, a wine strain *S. cerevisiae* ML01 was constructed, in which the malate transporter gene of *Schiz. pombe*, Sp*MAE*1 [21], and the *mle*A gene of *O. oeni* was integrated into its genome, allowing for efficient deacidification in the early stages of vinification [52]. Given the metabolic diversity found nowadays in yeasts isolated from fermentative environments, it is crucial to continue searching for strains that display positive technological traits, as an alternative to the engineering yeasts whose use still is limited in food industry.

## 4. De Novo Synthesis of Organic Acids 

**Figure 1:**
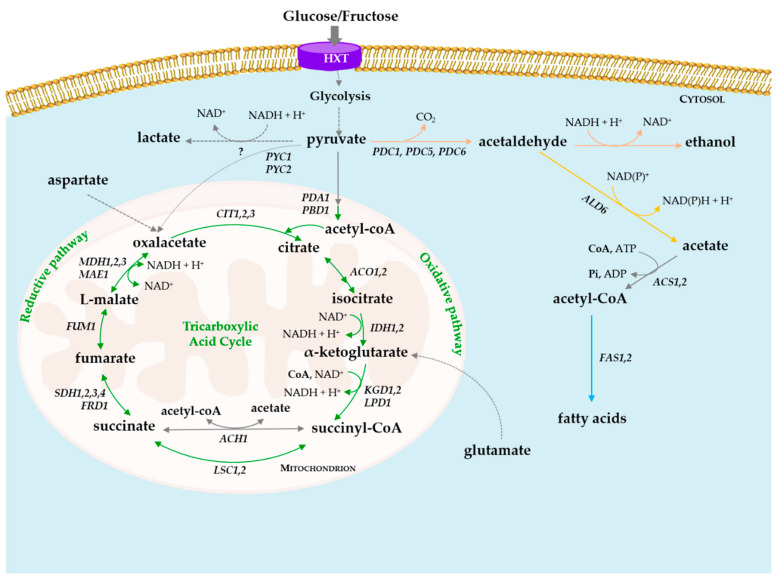
Schematic representation of the pathway of acids biosynthesis during fermentation. The reactions indicated are catalyzed by the enzymes, encoding by the following genes: *PDC1,5,6*—pyruvate decarboxylase; *ADH*—alcohol dehydrogenase; *ALD6*—aldehyde dehydrogenase; *ACS1, ACS2*—acetyl-CoA synthetase; *FAS1, FAS2*—Fatty Acid Synthetase; *PYC1,2*-cytosol—Pyruvate carboxylase; *PDA1, PBD1*—subunits of the pyruvate dehydrogenase complex; *CIT 1,2,3*—Citrate synthase; *ACO1,2*—Aconitase; *IDH1,2*—NAD(+)-dependent isocitrate dehydrogenase; *KGD1,2, LPD1*-α-ketoglutarate dehydrogenase complex; *ACH1*—Protein for SH-CoA transfer from succinyl-CoA to acetate; *LSC1,2*—subunits of succinyl-CoA ligase; *SDH1,2,3*—subunits of succinate dehydrogenase; *FRD1*—fumarate reductase; *FUM1*—fumarase; *MDH1*—mitochondrial malate dehydrogenase; *MAE1*—mitochondrial malic enzyme.

In addition to the changes in pre-existing acids, others are produced as a result of the metabolic activity of yeasts (Figure 1). Succinic acid is present in only trace amounts in grapes, but its concentration is higher in wines, 0.2 up to 3 g L^−1^ [53], as a result of yeast metabolism [1]. Free succinic acid imparts an “unusual salty, bitter taste in addition to its sourness“ [54], while its ethyl ester is associated with storage and wine aging. Succinic acid formation is dependent on the yeast strain genetic background [55,56], aeration conditions [55], fermentation temperature and chemical composition of the growth medium [53], particularly nitrogen availability and the source [56]. It is typically formed during the exponential growth phase [53,56] and, to a lesser extent, during the stationary phase [57]. Theoretically, succinic acid is formed in *S. cerevisiae* by four main pathways: (1) the reductive pathway of the TCA cycle; OAA formed via carboxylation of pyruvate (Pyc1,2) is reduced to l-malate (Mdh1), which after the loss of one water molecule, is converted to fumarate, and then reduced to succinate through Frd1; (2) the oxidative pathway of the TCA cycle, involving the oxidative decarboxylation of α-ketoglutarate, through a Kgd1,2, leads to succinate; (3) through the glyoxylate cycle, in which isocitrate is split into glyoxylate and succinate by the Icl1,2; and (4) from amino acid catabolism namely aspartate and glutamate [10]. In this case, OAA, produced from transamination of aspartate, is reduced to malate through mitochondrial or cytosolic aspartate aminotransferase, encoded by *AAT1* [58] or *AAT2* [59], respectively. *AAT2* appears to influence yeast fermentation duration under low-nitrogen conditions in a chemically defined grape juice [60]. Glutamate is converted into ammonia and α-ketoglutarate through Gdh1,2 [61], and then, through the GABA shunt pathway, converted into succinate by the concerted action of three enzymes: glutamate decarboxylase, GABA aminotransferase, and succinate-semialdehyde dehydrogenase [62]. In a chemostat culture, under limited-glucose concentration, *S. cerevisiae* CEN.PK113-1A produces higher concentrations of succinate under anaerobic than under fully aerobic conditions [63], suggesting that strict anaerobic conditions are not achieved in such chemostat culture system [64]. In batch-culture in YPD, with high-sugar concentration (150 g L^−1^) and under aerobic conditions, a *KGD1* gene-disrupted mutant produced lower amounts of succinate while *SDHl* gene-disrupted mutant produced more succinate than the wild type strain [65]. Similar findings have been reported in *KGD1* gene-disrupted mutant that produced less succinate during fermentation of a synthetic grape juice media with 200 g L^−1^ of glucose and 300 mg L^−1^ of glutamate as the only nitrogen source [66]. These results indicate that, under a high glucose concentration (150 to 200 g L^−1^), the majority of succinic acid is formed via the oxidative direction of the TCA cycle through Kgd1,2, which promotes the oxidative decarboxylation of α-ketoglutarate to succinic-CoA, which is converted to succinate [65,66]. Specific activities of α-ketoglutarate dehydrogenase and succinyl-CoA synthetase are markedly affected by large quantities of glutamate, glutamine, and/or threonine in the fermentation medium [56,66]. Under low nitrogen, yeast cells most likely produce succinic acid via the reductive branch of the TCA cycle to balance the NADH/NAD^+^ ratio [10]. In sake fermentation, it is formed by the oxidative pathway at the early phase and by a reductive direction in the later stages [65]. Altogether, results confirm that succinate could be synthesized through both directions, depending on the nitrogen source used [66].

Acetic acid is quantitatively the most important volatile fatty acid produced by yeasts [67]. It is mostly formed during the exponential growth phase, ranging from 100 to 300 mg L^−1^ [3]. The production is variable with the yeast strain used [68,69], fermentation temperature [70] and chemical composition of the grape juice, particularly the levels of sugar, vitamins [10], and nitrogen concentration [71,72]. Acetate is produced by the irreversible oxidation of acetaldehyde to acetate by Ald6, a cytosolic enzyme activated by Mg^2+^, use NADP^+^ as the preferred coenzyme, and is active during fermentation. Therefore, acetic acid is most produced in the cytosol through a set of reactions, which involves the enzymes pyruvate decarboxylase, acetaldehyde dehydrogenase, and acetyl-CoA synthetase [73,74].

The acetic acid formed at the beginning of fermentation via acetyl-CoA is used at later stages by yeasts [3]. High activity of acetyl-CoA synthetase was detected when no acetic acid was formed in a *S. cerevisiae* strain [64]. The synthesis of acetyl-CoA from acetic acid increases after overexpressing the *ACS2,* a gene that confers acetic acid resistance, indicating that the consumption of the acid is Acs2-mediated and can be seen as a mechanism of detoxification [75].

Natural *S. cerevisiae* strains produce only traces of d-lactic acid during alcoholic fermentation due to the lack of an efficient lactic acid pathway. Levels up to 100–500 mg L^−1^ have been reported in final wines [10], being this formation associated with a defective pyruvate decarboxylase activity. A lack of thiamine favors the production of lactic acid, suggesting, in this case, that NADH is oxidized by the reduction of pyruvate rather than by the reduction of acetaldehyde to ethanol [54]. Thus, the presence of high amounts of lactic acid in wine has been seen as indicative of bacterial activity. One of the few yeast strains producing lactic acid is *Lachancea thermotolerans*, (previously *Kluyveromyces thermotolerans*), [76,77]. The species shows relatively extensive fermentative ability, low production of acetic acid and formation of lactic acid [78]. This trait has prompted the use of *L thermotolerans* strains as adjuncts of *S. cerevisiae*, either in mixed or sequential fermentations, as a tool to mitigate the effects climate change in warm viticultural regions due to its ability to increase wine acidity and thus surpass the lack of freshness of wines produced from these grapes [79]. 

A very small amount of pyruvic acid remains in wines at concentrations up to 100 mg L^−1^ [10]. Concentration greater than about l00 mg L^−1^ is indicative of a cofactors thiamine pyrophosphate (TPP) deficiency [54] or a suboptimum nitrogen supply [10]. High amount of organic acids—namely fumaric, pyruvic, and α–ketoglutaric—and small amounts of ethanol and acetic acid has been detected in a non-*Saccharomyces* strain of *Starmerella bacillaris*, grown in synthetic medium with high-sugar concentration [80]. Pyruvate flux out of yeast cells can be seen as a detoxification mechanism to prevent a decrease in cell pH from the excess pyruvate [54]. Pyruvate is an essential intermediate, being the precursor of many fermentation byproducts. In *S. cerevisiae*, three pyruvate decarboxylases isoforms (*PDC1, PDC5, PDC6*) are implicated in the decarboxylation of pyruvate to acetaldehyde and CO_2_. Part of the pyruvate not used by PDCs participates in the formation of acetic acid, lactic acid, 2,3-butanediol, diacetyl, and acetoin. 

Yeasts have the ability to produce and excrete several other oxo-acids—such as 2-α-ketoglutarate, 2-oxobutyrate, 2-oxoisovalerate, 2-oxo-3-methylvalerate, and oxoisocaproate—regarded as products of amino acids metabolism [10,54]. Oxo-acids can undergo oxidative decarboxylation in the presence of NAD^+^ to form acyl-CoA, proceeding the activity of acyl-coenzyme A: ethanol O-acyltransferase (Eht1), an enzyme that plays a minor role in medium chain fatty acids (MCFA)-ethyl ester biosynthesis by catalyzing the transference of fatty acyl groups from acyl-CoA to ethanol [81,82,83].

MCFAs such as hexanoic, octanoic, decanoic, and dodecanoic are typically identified in wine after fermentation [84]. Medium composition [85]—particularly the initial nitrogen concentration [84] and the timing of nitrogen addition to the fermenting media [72], temperature variation [86], and the strain of *S. cerevisiae* used [84]—are factors reported to influence the formation of fatty acids. MCFA are generally associated with non-agreeable sensory attributes [85,87] whereas their ethyl-esters provide pleasant fruity and floral notes to the wine [88,89]. The MCFAs, particularly octanoic and hexanoic, are synthesized via a fatty acid synthase complex, starting with two molecules of cytosolic acetyl-CoA, and one is irreversibly carboxylated to malonyl-CoA by the biotin-dependent enzyme acetyl-CoA carboxylase (*ACC1*) [90]. The assembly of both acetyl-ACP and malonyl-ACP results in acetoacetyl-ACP through the enzymatic complex fatty acid synthetase, encoded by *FAS1* and *FAS2* [91]. Malonyl-CoA is used in an iterative process, in which the fatty acid chain is progressively elongated, by adding two-carbon units at a time, repeating the cycle until the final length is completed. Ethyl-esters biosynthesis likely proceeds enzymatically by reactions involving the condensation of activated fatty acids (acyl-CoA) and ethanol [80,88]. Acyl-CoA is formed by the activation of fatty acids or by the oxidative decarboxylation of ketoacids [91]. The synthesis of the esters begins when the synthesis of the lipids ceases, suggesting that the synthesis of the lipids modulates the synthesis of the esters. 

The acyl-CoA: ethanol O-acyltransferase (Eeb1) and the ethanol hexanoyl transferase (Eht1) are responsible for the major part of MCFA-ethyl ester biosynthesis during fermentation [81,92]. A strong positive correlation between *EHT1* expression with the levels of ethyl esters of caproate, caprylate, and caprate has been reported [92,93], while the opposite has also been observed, i.e., the overexpression of *EHT1* or *EEB1* has not increased MCFA-ethyl ester content [80]. On other hand, the expression of *MGL2*/YMR210W, which encodes monoacyl-glycerol lipase, an enzyme with a minor role in MCFA-ethyl ester biosynthesis, similar to the Eeb1 and Eht1, was shown to be positively correlated with ethyl caprylate [94]. Despite the contradictory results about whether Eht1 does influence MCFA-ethyl ester biosynthesis, formation of such esters is regarded as a detoxification mechanism of yeast to eliminate the excess of acids [95]. 

There are a few reports on formation of malic acid from *S. cereviseae* isolated from cider and wine [54] and from a sugar-tolerant yeast *Zygosaccharomyces rouxii,* which produces high amounts of malic acid and occasionally also succinic acid, being such production stimulated by glutamate and malate, respectively [96,97]. The amount of malic acid produced is generally dependent on the yeast strain, being favored by a high concentration of carbohydrates (200–300 g L^−1^), relatively high pH (pH 5.0), and limited nitrogen availability, 100 to 250 mg L^−1^ [10]. Overexpression of *MDH2* inhibits the production of malic acid in strains growing in high nitrogen medium [94,96]. A cytosolic reductive pathway of l-malic acid biosynthesis and accumulation has been previously demonstrated in *S. cerevisiae* [94,96], it arises from the reduction of OAA, with the concomitant oxidation of NADH. 

Citramalic acid (2-hydroxy-2-methylbutanedioic acid) has a structure similar to that of malic acid, with an extra methyl group on it. It has been reported as a byproduct of *Saccharomyces* species and first detected in wines in concentrations ranging from 25–173 mg L^−1^, [54], dependent on the yeast strain genetic background [96], medium composition, particularly the nitrogen source [54]. Yeast produce citramalic and dimethylglyceric acid in concentrations up to 300 or 600 mg L^−1^, respectively [3]. These compounds show minor organoleptic impact in wine [3], while addition of citramalic acid strengthened the saltiness and umami, and weakened the sourness and bitterness in Japanese sake [98]. 

The acids produced by yeast may act as substrates on the biosynthesis of esters, compounds that contribute to a pleasant fruity bouquet. In wine, there are mostly two type of esters: the acetate esters and ethyl esters. Acetate esters results from the condensation of acetyl-CoA with ethanol or higher alcohols by acetyltransferases, encoded by the *ATF1* and *ATF2* genes [99,100]. The ethyl esters, produced at much lower levels than acetate esters, result from the condensation of the acid group of a short-chain or a MCFA with the alcohol group of ethanol [99,100].

*S. cerevisiae* produce all of these organic acids during growth but it only excretes them, if any, in very small amounts, therefore industrial utilization of this trait is not practical [10]. Microbial production of carboxylic acids has recently gained major interest for replacing petroleum-based chemicals, with a great investment from academia and industry to continue looking for more effective microorganisms. *S. cerevisiae*, a workhorse of industrial microbiology, offers several advantages: it is a fast-growing organism even in minimal medium at low pH, it utilizes a wide range of carbon sources, it is generally recognized as safe, and it has been extensively genetically characterized [101], which makes it a highly attractive organism for metabolic engineering. Metabolic engineering, by increasing the expression of key pathway genes, along with deletion of competing pathways, has proven to be quite effective for enhancement of carboxylic acids production [102], such as succinic acid [103,104] and lactic acid [105,106]. Despite the progress in metabolic engineering in *S. cerevisiae* to increase chemicals production, there are still problems to be solved, particularly the high cost of the development of a robust cell factory and all of the optimization processes to increase yield and productivity.

## 5. Lactic Acid Bacteria of the Wine

Lactic acid bacteria (LAB) comprise a group of Gram-positive, non-spore forming, microaerophilic, or anaerobic bacteria that produces lactic acid as a major end-product from fermentative metabolism of carbohydrates. LAB are typically catalase negative, very fastidious from a nutritional point of view, largely with complex nutritional requirements, aero-tolerant, and acid tolerant. Current classification encompasses LAB to the phylum *Firmicutes*, Class *Bacilli*, Order *Lactobacillales*, which includes *Lactobacillaceae* or *Leuconostocaceae* among other families [107]. Analysis of 16S rRNA gene similarity combined with the analysis of the carbohydrate fermentation profile divide LAB into three groups, the obligate homofermentative, that almost exclusively produces lactic acid through glycolysis or the EMP pathway; the obligate heterofermentatives that use hexoses and pentoses through the pentose phosphoketolase pathway (hexose monophosphate shunt/6-phosphogluconate pathway) and produce equimolar amounts of lactate, CO_2_, and ethanol or acetate [108]; and the facultative heterofermentative that use hexoses through glycolysis and pentoses through the pentose phosphoketolase pathway [107,108].

LAB are among the most important groups of microorganisms used in the food industry and are the most common microbes employed as probiotics [109]. Wine-associated LAB comes from grapes and vineyards, as well as from wine cellars and equipment. They include members of the genera *Leuconostoc, Oenococcus, Pediococcus*, *Lactobacillus*, and *Weissella*. The latter genus emerged after the analysis of the rDNA of *Lc. paramesenteroides*, reclassified as *W. paramesenteroides*, and five heterofermentative *Lactobacillus, L. confusus, L. halotolerans, L. kandleri, L. minor*, and *L. viridescens*, were included in this genus [108]. Some of the genera *Pediococcus* spp. and *Lactobacillus* are widely associated with wine spoilage or with producing toxic substances, such as biogenic amines and other undesirable metabolites [67]. All wine-LAB are capable of using malic acid [67], but *Oenococcus oeni* (formerly *Leuconostoc oenos*, [110]) is probably the best adapted species to resist to the harsh wine conditions, and remains the most preferred species for inoculation of wines in which MLF is desired [111,112,113,114]. *Pediococcus* spp., once considered spoilage agent, is now shown to be potentially useful for producing wines from musts with pH above 3.5. In addition, the panoply of enzyme activities exhibited by some of this homofermentative bacterium compared with *O. oeni*, may give winemakers the chance to develop new wine styles [112]. However, the most problematic wines are those with the lowest pH values, in which the occurrence of MLF remains unpredictable. This process may take place during alcoholic fermentation, but more often, it occurs later, usually within the first year, or sometimes not at all [1,3]. Stimulation of MLF by inoculation with selected strains of *O. oeni* available on the market is not always successful. This difficulty can be overcome by the use of indigenous starter-cultures well-adapted to the conditions of the specific wine-producing area. 

Species other than *O. oeni* are used as starter-cultures for wine deacidification. Some strains of *Lactobacillus plantarum* are able to survive or even grow under the harsh conditions of the wine environment—i.e., high ethanol, low pH, and the presence of sulfite [115,116,117]—and to conduct MLF just as efficiently as *O. oeni* [112], properties that make them suitable for MLF starter-cultures [108]. A new highly concentrated *Lb. plantarum* starter is recently available in the market [118]. Despite the good alcohol tolerance, that strain is homo-fermentative, for hexoses metabolism, which makes its application with no risk of volatile acidity production [118]. A panoply of enzymatic activities—such as glycosidase, protease, esterase, and citrate lyase—has been reported in different strains of *Lb. plantarum* [119] and in *O. oeni*, which can result in favorable modification of the sensory profile of the wines [120,121,122]. Additionally, numerous strains produce bacteriocins, such as plantaricin in *Lb. plantarum*, which would assist them in inhibiting spoilage bacteria [123]. Another remarkable trait is its ability to degrade biogenic amines [124], traits very remarkable when safety is the goal. Biogenic amine-degrading microorganisms can be a useful tool to reduce the levels of biogenic amines in wines [125]. Ensuring the success of MLF largely depends on the LAB strain used and other factors, namely, its geographical origin and adaptability to the winemaking process. 

## 6. Role of Lactic Acid Bacteria on Acid Modulation

**Figure 2:**
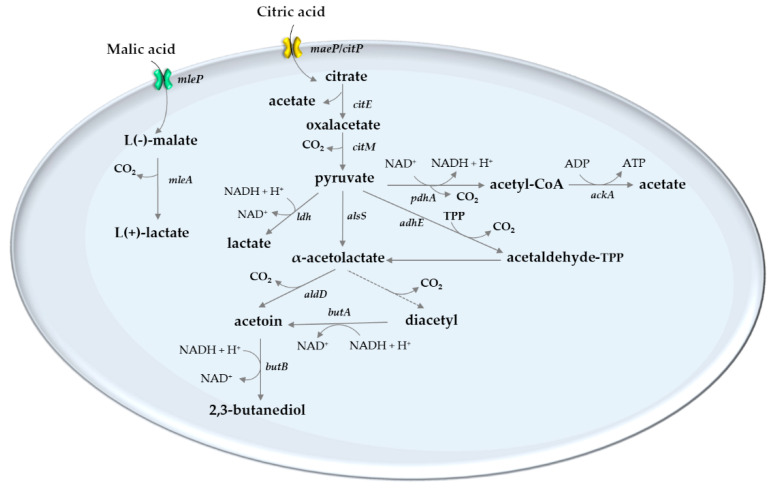
Schematic representation of the pathway for citrate and malate metabolism by lactic acid bacteria. The reactions indicated are catalyzed by the following enzymes: *mleP*—malate permease; *mleA*—malolactic enzyme; *citP*/*maeP*—putative citrate permease; *citE*—citrate lyase; *citM*—oxaloacetade decarboxylase; *ldh*—lactate dehydrogenase; *pdh*—pyruvate dehydrogenase; *ackA*—acetate kinase; *alsS*—α-acetolactate synthase; *alsD*—α-acetolactate decarboxylase; *adhE*—acetaldehyde dehydrogenase; *butA*—acetoin dehydrogenase; *butB*-2,3—butanediol dehydrogenase; TPP—thiamine PPi.

The ability to use malic and citric acids is widespread among LAB, which are readily metabolized anaerobically with consequent flavor changes (Figure 2). The conversion of l-malic acid to l-lactic result from one of three pathways: most LAB converts the C4 dicarboxylic acid L(-) malic acid into the C3 monocarboxylic acid L(+) lactic acid and CO_2_ through a NAD^+^ and Mn^2+^-dependent malolactic enzyme (MLE), without any free intermediates [126]. Most use MLE instead of a malic enzyme (ME), except *Lactobacillus casei* [127] and *Enterococcus faecalis* (formerly *Streptococcus faecalis)*, whose enzymatic properties are almost identical to those of other malic enzymes previously described [128]. Malic and malolactic enzymes are distinct at the phylogenetic level, except for malic enzymes from yeast and *Escherichia coli*, which were closer to the MLE than the other ME [129]. 

Malate utilization by *Enterococcus faecalis* involves ME (l-malate: NAD+ oxidoreductase (decarboxylating), E.C. 1.1.1.39), which is induced by malate, uses NAD+ as a cofactor and requires Mn ^(+2)^ or Mg ^(+2)^ for optimal activity, followed by l-lactate dehydrogenase acting on converting pyruvate and NADH to l-lactate and NAD^+^ [126]. *Lb. casei* appears to be the only one [130,131] in which l-malate degradation is attained through both MLE and ME pathways. The utilization of l-malate through MLE cannot sustain bacteria growth, while the ME pathway enables these species to grow on l-malate as a carbon source [131]. Another exception is *Lb. fermentum*, which metabolizes l-malic acid to d- and l-lactic acid, acetate, succinate, and carbon dioxide [119]. MLF in *O. oeni* is an energy-producing mechanism involving electrogenic monoanionic l-malate uptake, intracellular decarboxylation of the acid by MLE, and the efflux of L-lactic acid plus carbon dioxide [132,133]. Genetic organization of the malolactic locus in *O. oeni* demonstrated that it contains the genes encoding the MLE (*mle*A), the l-malate carrier protein (*mle*P) apparently transcribed in the same operon, and the *mleR*, the LysR-type regulatory protein, transcribed in the opposite direction and regulator in the expression of *mleA,P* [134], characteristics that makes it particularly effective for degradation of the acid.

Citric acid utilization with the formation of metabolic end products such as diacetyl and acetate has impact on aroma and on wine stability. In wines, citrate exists in concentrations below 500 mg L^−1^ [10] and can be utilized by a limited number of LAB [10,110,135]. Diacetyl, acetoin, 2-3-butanediol, acetaldehyde [135], and acetate [136] are end-products of sugar or citrate metabolism. Citrate is metabolized by *O. oeni,* in a pH- and temperature-dependent manner, and the major end products were acetate and diacetyl. The degradation of citric acid start at the same time as the malic acid degradation, but at a much slower rate [136], corroborating previous findings that at the end of the MLF, there still exists some citric acid, up to 150 mg L^−1^ or sometimes even more [3]. Most of the knowledge about the biochemical pathway of citrate utilization by LAB is derived from dairy-LAB [137]. The key enzymes of citrate metabolism are: (1) citrate permease, an enzyme with a crucial role in the uptake of citrate into the cell, which action is strongly dependent on the pH (pH 5–6 in *Lactococcus lactis* [135], pH 4.0 in *Lb plantarum* [138] and even lower in *O. oeni* [139]); (2) citrate lyase enzyme, through which citrate is split into acetate and OAA [135]; and (3) OAA-decarboxylase, which decarboxylates OAA to pyruvate in *O. oeni* [139], as it has been found in *Lactococcus* spp [136]. The absence of citrate lyase in some strains of *Lb. plantarum* has been reported [138], a trait that clearly makes this species particularly useful, since it may not boost an increase in volatile acidity after MLF. In contrast, some strains of this species produce succinate from citrate by the reductive TCA cycle, which utilizes citrate lyase, malate dehydrogenase, fumarase, and fumarate reductase [139] that could cause the titratable acidity to increase. As mentioned above, *Lb. plantarum* is a facultative heterofermentative LAB that exhibits a homolactic fermentation pattern during growth on glucose leading to pyruvate, which is next converted to roughly equimolar amounts of d- and l-lactate by the activities of stereospecific lactate dehydrogenase enzymes [108]. Consistently, its genome encodes all enzymes required for the glycolysis and phosphoketolase pathways [140]. In addition to the lactate dehydrogenase genes, *ldh*L and *ldh*D, the chromosome also encodes two other putative genes for lactate dehydrogenase and a relatively large number of other pyruvate scattering enzymes that are foreseen to catalyze the production of other metabolites, such as formate, acetate, ethanol, acetoin, and 2,3-butanediol [140]. Some findings have corroborated previous reports that some strains of *Lb. plantarum* produce lactate, acetoin, and/or acetate when incubated in a citrate-containing medium [138], indicating that succinate production is not a phenotype shared by all *Lb. plantarum* strains. In short, pyruvate can be reduced to lactate by lactate dehydrogenase or decarboxylated in the presence of the coenzyme TPP, resulting in the formation of hydroxyethyl-TPP (active acetaldehyde). The condensation of a pyruvate molecule with acetaldehyde-TPP leads to the formation of α-acetolactic acid, which can be oxidatively decarboxylated to diacetyl. Diacetyl and acetoin can be produced by α-acetolactate but, while acetoin is formed by either the nonoxidative decarboxylation of α-acetolactic acid or the reduction of diacetyl, diacetyl results from a nonenzymatic oxidative decarboxylation [108,135]. 2,3-butanediol arises from the reduction of acetoin and contrary to diacetyl, an important flavor compound, it has no special sensory impact in wine. 

Also pyruvate, mostly produced by yeasts during alcoholic fermentation, can be oxidatively decarboxylated by LAB, with phosphate entry, to acetyl phosphate, which can be converted into acetate and ATP or, at a high ratio of intracellular NADH/NAD+, to acetaldehyde, which is then reduced to ethanol [1,3]. Constitutive overproduction of the pyruvate oxidase gene (*poxB*) in *L. plantarum* revealed the predominant role of pyruvate oxidase in acetate production under aerobic conditions [141].

LAB are fastidious organisms, requiring complex organic nitrogen sources, such as amino acids. The activity of decarboxylase enzymes has advantages since it allows for an increased pH, which makes the harsh environment more favorable for LAB survival. In this way, some species have the ability to utilize a large number of amino acids with a high risk of histamine and tyramine production during MLF [142]. Biogenic amines are low molecular weight organic bases with aliphatic, aromatic, and heterocyclic structures generally found in fermented food [143]. Histamine content in wines can be up to 30 mg L^−1^, depending on the type of wine; as expected, white wines have lower values than red wines [144]. The use of amine-degrading starters seems to be a promising tool to reduce the biogenic amine content in fermented foods or in beverages [124]. 

Other minor acids can undergo small changes during the winemaking process. Degradation of lactic acid in the presence of citrate and glucose has been reported in *Lb. plantarum*. In contrast, degradation of succinic acid was not detected under either anaerobic or aerobic conditions in LAB. Gluconic acid can be metabolized by *Lactobacillus* and *O. oeni* by the hexose monophosphate pathway, leading to lactic acid, acetic acid, and/or ethanol and CO_2_ [108]. Also, α-ketoglutaric acid can be decarboxylate into semi-aldehyde-succinate by *O. oeni*, being reduced to 4-hydroxybutyrate or oxidized to succinate [145]. The metabolic activity of LAB is not circumscribed to the utilization of the organic acids initially existing in grapes. Therefore, wines must attain complete dryness before the occurrence of MLF because the potential use of sugars by LAB may lead to the formation of undesirable metabolites that depreciate wine aroma and/or to the formation of high concentrations of acetic acid, which should be kept at the lowest possible level. 

## 7. Conclusions

l-tartaric and l-malic acids account for over 90% of organic acids content in grape juice. The progressive reduction in malate content throughout ripening is due to the concerted action of the cytosolic MDH and the NADP+-ME, involved in both malate synthesis and degradation, while tartaric acid content remains relatively constant. Malic acid is easily degraded by most wine microorganisms. *S. cerevisiae* unveil a weak ability to metabolize it. In contrast, *Schiz. pombe* can efficiently degrade malic acid since in addition to an efficient transport system, an effective cytosolic ME acts in its oxidative decarboxylation into pyruvate. Other organic acids, as succinate, acetate, among others are produced de novo by yeasts during fermentation. The results currently obtained suggests that succinate is formed in *S. cerevisiae* through the oxidative or by the reductive direction of the TCA cycle, depending on the fermentation stage and on the nitrogen source used. The acids produced by yeast may act as substrates on the biosynthesis of esters, as it is the case of the MCFA which are generally associated with non-agreeable sensory attributes, whereas their ethyl-esters provide pleasant fruity and floral notes to the wine. Natural *S. cerevisiae* strains produce little or no lactic acid, suggesting the lack of the enzymes lactate dehydrogenases. This trait has prompted the use of *L. thermotolerans* strains either in mixed or sequential fermentations, as a tool to mitigate the effects of climate change in warm vinicultural regions.

In contrast to what has been observed in yeasts, the ability to use malic and citric acids is widespread among LAB, which are readily metabolized with consequent flavor changes. Acetate, lactate, diacetyl, acetoin, and 2-3-butanediol are end-products of citrate metabolism. Species other than *O. oeni*, namely strains of *Lb. plantarum* and *Pediococcus* spp., once considered spoilage agents, are now shown to be potentially useful to give winemakers the chance to develop new wine styles, given the panoply of enzymatic activities in such strains.

In conclusion, current knowledge suggests that is wise the use of other non-conventional yeasts, as adjuncts of *Saccharomyces*, in co- or sequential fermentation, for enhancing the complexity of wine aroma. These yeasts can contribute to less alcoholic or more acidic wines, interesting features to address the problem of unbalanced grapes that exist in contest of global warming. LAB can be seen as a group of very willing bacteria that may add small amounts of metabolic byproducts, which may improve wine flavor. The potential impact of both group of microorganisms in wine quality largely justifies to use a rigorous and meticulous program for selection of strains better adapted to the particular conditions of a specific wine-producing region.
